# *Cerebrospinal Fluid Research: *A new platform for dissemination of research, opinions and reviews with a common theme

**DOI:** 10.1186/1743-8454-1-1

**Published:** 2004-12-10

**Authors:** Hazel C Jones

**Affiliations:** 1 Editorial Office, Gagle Brook House, Chesterton, Bicester, Oxon OX26 1UF, United Kingdom

## Abstract

*Cerebrospinal Fluid Research *aims to provide a specialist platform for high quality articles on all aspects of the cerebrospinal fluid (CSF), bringing together experts working in the field and promoting synthesis and dialogue. This, launch Editorial provides an overview of the field, some history, and highlights some of the journal policies.

Cerebrospinal Fluid Research is an open access, online journal that publishes manuscripts on cerebrospinal fluid (CSF) in health and disease in the broadest sense. The CSF, its composition, circulation and absorption play vital roles in normal and abnormal brain function. The fluid within the CSF system is important for normal chemical signaling, physical and chemical buffering, and for neurodevelopment. In disease states, the CSF impacts on neurodevelopmental disorders such as hydrocephalus and neural tube defects, brain inflammation, brain injury and repair, normal pressure hydrocephalus and neurodegenerative diseases such as Alzheimer's, Parkinson's and multiple sclerosis. The CSF can be used as a tool for diagnosis, through composition analysis, and as a window for drug delivery to the brain.

Fluid within the brain cavities was known of in ancient times. Hippocrates in the 4^th ^century B.C. recorded the presence of fluid and is thought to have tapped the ventricles in a patient with hydrocephalus. Subsequently, in the 2^nd ^century A.D., Galen described the ventricles and his dogma that the ventricles contained a gaseous vital spirit, lasted for over 1000 years. The return to the fluid hypothesis occurred in 1543 with Vesalius who made detailed observations of the anatomy and noted the presence of a watery humor. Further studies in the 17^th ^and 18^th ^centuries by Valsalva, Haller, and Contugno elaborated and extended this knowledge. Magendie in 1825 made chemical and physiological studies on the fluid and coined the name *liquide cephalo*-*rachidien *or fluid cerebrospinal. He saw pulsatile movement and concluded the fluid was under positive pressure. Later in the 19^th ^century the anatomists Key and Retzius made extremely detailed studies of the cavities and the membranes of the brain, and provided a foundation for many 20^th ^century investigations starting with Dixon and Haliburton, 1913 [1], Dandy and Blackfan, 1914 [2] and Weed 1935 [3]. At this stage it was known that the fluid originates in the choroid plexus and circulates throughout the internal cavities and external spaces to the venous sinuses. It was also shown by dye studies that there was a 'barrier' for movement of substances between the blood and the brain (blood-brain barrier) and between the blood and CSF (blood-CSF barrier).

Physiological studies began in the 1950's with numerous investigations led by Hugh Davson, the 'father' of CSF physiology [4]. It was demonstrated that the CSF was not a plasma ultrafiltrate but a secreted fluid under homeostatic control with its own unique composition for electrolytes, small non-electrolytes and proteins. Hugh Davson was a remarkable scientist who aided and encouraged many collaborating researchers to work in the field of CSF.

Particular milestones in the latter half of the 20^th ^century include the measurement and control of CSF secretion by Pappenheimer et al [5], and uptake functions for the choroid plexus by Welch [6] and Pollay and Davson, [7], the demonstration of tight junctions at the endothelium and choroid epithelium by Brightman and Rees [8], and the effect of inhibitors on secretion by Davson and Segal [9]. Further studies have brought us to understand that the choroid plexus is regarded as a complex secretory, regulatory, and absorptive organ. The CSF is no longer considered to be only a cushion for the brain but a multifunctional organ with homeostatic, hormonal and signaling mechanisms that have important functions in health, and particularly in neurological diseases. This is an active and fast growing field containing researchers in many disciplines. However, to date, the subject area does not have a specialist platform and articles are published in a variety of different journals. We invite you to help us to bring the field under one umbrella by submitting your manuscripts online to *Cerebrospinal Fluid Research *[10]. The journal will provide a comprehensive medium, offering quality peer-review of manuscripts on all aspects of CSF.

*Cerebrospinal Fluid Research *is supported by an international Editorial Board [11]. Each manuscript will be reviewed by a member of the Board or, where appropriate, allocated to external reviewers. At least two reviewers will be sought for each manuscript; a third will be approached where there is a significant difference in opinion. Reviewers can choose whether to remain anonymous. Authors will be asked to provide a list of suggested reviewers on submission of their manuscript.

*Cerebrospinal Fluid Research *will consider the publication of research articles, reviews, commentaries, book reviews and meeting proceedings. All articles will be published immediately upon acceptance (after peer review) and listed in PubMed. The intention is to publish, as supplements, the proceedings of scientific meetings in relevant subject areas.

*Cerebrospinal Fluid Research's *Open Access policy changes the way in which articles are published. First, all articles become freely and universally accessible online, and so an author's work can be read by anyone at no cost. Second, the authors hold copyright for their work and grant anyone the right to reproduce and disseminate the article, provided that it is correctly cited and no errors are introduced [12]. Third, a copy of the full text of each Open Access article is permanently archived in an online repository separate from the journal. *Cerebrospinal Fluid Research's *articles are archived in PubMed Central [13], the US National Library of Medicine's full-text repository of life science literature, and also in repositories at the University of Potsdam [14] in Germany, at INIST [15] in France and in e-Depot [16], the National Library of the Netherlands' digital archive of all electronic publications.

Open Access has broad benefits for science and the general public. Most importantly, authors are assured that their work is disseminated to the widest possible audience, given that there are no barriers to access their work. In addition, authors are free to reproduce and distribute their work, for example by placing it on their institution's website. Furthermore, there is evidence that free online articles are more highly cited because of their easier availability [17] and publicly funded research will become accessible to all taxpayers (not just those with access to a library with a subscription). As such, Open Access could help to increase public interest in, and support for, research. Note that this public accessibility may become a legal requirement in the USA if the proposed Public Access to Science Act is made law [18]. Resource-poor countries (and institutions) will be able to read the same material as wealthier ones providing they have access to the internet [19].

This is an exciting opportunity to disseminate our science in the new world wide medium of electronic publishing. *Cerebrospinal Fluid Research *looks forward to receiving your submissions.
